# *Schisandra chinensis* (Turcz.) Baill. polysaccharide inhibits influenza A virus *in vitro* and *in vivo*

**DOI:** 10.1002/2211-5463.13690

**Published:** 2023-09-01

**Authors:** Jing Qi, Chaoxiang Lv, Jin Guo, Yuanguo Li, Mingwei Sima, Rongbo Luo, Haiyang Xiang, Xianzhu Xia, Yifa Zhou, Tiecheng Wang

**Affiliations:** ^1^ College of Life Sciences Northeast Normal University Changchun China; ^2^ Changchun Veterinary Research Institute, Chinese Academy of Agricultural Sciences China; ^3^ The Research Center for Preclinical Medicine Southwest Medical University Luzhou China; ^4^ College of Life Sciences Shandong Normal University Jinan China; ^5^ College of Basic Medicine Changchun University of Chinese Medicine China

**Keywords:** antiviral, immunomodulation, inflammation, influenza, polysaccharides

## Abstract

Influenza virus is prone to seasonal spread and widespread outbreaks, which pose important challenges to public health security. Therefore, it is important to effectively prevent and treat influenza virus infection. *Schisandra* polysaccharide (SPJ) is a polysaccharide derived from the fruit of *Schisandra chinensis* (Turcz.) Baill. In this study, we evaluated the antiviral activity of SPJ *in vitro* and *in vivo*, especially against influenza A virus (IAV) infection. By analyzing SPJ structure and monosaccharide composition, the molecular weight of SPJ was determined to be 115.5 KD, and it is composed of galacturonic acid (89.4%), rhamnose (0.8%), galactose (4.4%), arabinose (3.8%), and glucose (1.7%). Immunofluorescence analysis showed that SPJ treatment reduced the positive rate of viral nucleoproteins in cells, indicating that the compound had an inhibitory effect on influenza virus replication. Furthermore, SPJ therapy improved the survival of infected mice. Lung virus titer assays indicated that SPJ treatment significantly reduced viral loading in the lung tissue of infected mice and alleviated the pathological damage caused by influenza virus infection. Moreover, SPJ reduced cytokine expression during influenza virus challenge. In conclusion, SPJ has anti‐influenza virus effects and may have potential as an anti‐influenza drug candidate in further clinical studies.

## Graphical abstract

*Schisandra* polysaccharide (SPJ) is an acidic polysaccharide derived from ripe fruits of *Schisandra chinensis*. In our study, SPJ treatment not only inhibited influenza virus replication *in vitro*, but also prolonged the survival time and alleviated pathological damage of mice infected with influenza virus. SPJ also reduced the inflammatory response after infection, indicating its potential as a candidate drug for further research.

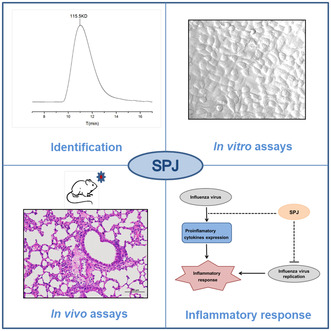

**Abbreviations**
BSA: bovine serum albumin
DAPI: 4′,6‐diamidino‐2‐phenylindole
DMEM: Dulbecco's Modified Eagle Medium
DMSO: dimethyl sulfoxide
ELD_50_
: embryo lethal dose 50
IFN: interferon
IL: interleukin
MDCK: Madin–Darby canine kidney
mLD_50_
: mouse lethal dose 50
MTT: 3‐(4,5)‐dimethylthiahiazo(‐z‐y1)‐3,5‐di‐phenytetrazoliumromide
SPJ: *Schisandra* polysaccharide
TNF: tumor necrosis factor

Influenza viruses in the *Orthomyxoviridae* family are primarily divided into four types: A, B, C, and D [1]. Influenza A virus (IAV) is highly variable and transmissible, readily causing seasonal epidemics [2]. Human infection with influenza virus can induce severe pneumonia, acute respiratory distress syndrome, septic shock, and a significant mortality rate [3]. This pathogen poses a tremendous threat to social public health. In recent years, viruses have frequently spread among species, leading to new outbreaks of disease, including H1N1 seasonal influenza virus [4], and H5N8 avian influenza virus [5]. Therefore, it is of great importance to identify novel anti‐influenza virus drugs.

Medicinal plants have been widely used to treat various infectious and non‐communicable diseases. Natural plant polysaccharides are polymeric carbohydrate molecules composed of long‐chain monosaccharide units with various biological activities, including antitumor [6] anti‐inflammatory [7], immunomodulatory [8], antioxidant [9], and antiviral effects [10]. Previous studies have reported the inhibitory effect of plant polysaccharides on viral replication in humans and animals, including *aloe vera* polysaccharide [11], *isatis* root polysaccharide [12], and *kelp* polysaccharide [13]. These findings not only broaden the efficacy range of plant polysaccharides, but also provide a new approach for the development of antiviral drugs.

*Schisandra chinensis* (Turcz.) Baill. polysaccharide (SPJ) is derived from its ripe fruits, which belongs to the *Magnoliaceae* plant, and contains various compounds, such as lignans, volatile oils, polysaccharides, organic acids, fatty acids, and proteins [14]. It has various functions, including immune regulation [15], liver protection [16], antifatigue effects [17], antioxidation [18] and antitumor activities [19], anti‐inflammatory [20], and reproductive system improvement [21]. Besides, as a component of hugan pill, *S. chinensis* can be clinically used to treat chronic hepatitis and early cirrhosis [22]. The polysaccharide of *S. chinensis* has been reported to improve liver injuries [23], reduce serum immunoglobulin and TNF‐α and IL‐2 levels caused by cyclophosphamide [24], and treat chronic cough [25], suggesting that it has the effect of activating the body's immunity and immune regulation. Modern research on the pharmacological effects of SPJ has focused on anti‐inflammatory, antioxidation, antifatigue, and immunological and antitumor effects. However, there are few reports on its antiviral activity.

In this study, we initially evaluated the antiviral activity of SPJ against influenza viruses *in vitro*, and then assessed its effect on IAV infection by a mouse model. Our investigations have revealed that SPJ reduces the expression of IL‐6, TNF‐α, IL‐10, and other factors, and that SPJ treatment reduces viral load in the lung tissues of influenza virus‐infected mice. Importantly, administration of SPJ ameliorates histopathological damage caused by influenza virus infection. These findings suggest that SPJ is a potential candidate for anti‐influenza therapy.

## Materials and methods

### Virus, mice, antibodies, and animal ethics statement

Human influenza virus H1N1 (A/Changchun/01/2009(H1N1)) and H3N2 (A/Sydney/5/97(A/H3N2)), as well as H1N1‐UI182, which is the mouse‐adapted strains of human influenza virus H1N1 (A/Changchun/01/2009(H1N1)), were stored at the Changchun Veterinary Research Institute, Chinese Academy of Agricultural Sciences. Six‐to‐eight‐week‐old female BALB/c mice (18–20 g) were obtained from Vital River Laboratory Animal Technology Co., Ltd. (Beijing, China). The mouse monoclonal antibody vNP (ab128193) was purchased from Abcam Company (Cambridge, UK). All mice were handled in accordance with the welfare and ethical guidance of Chinese laboratory animals (GB14925‐2001), and the study was approved by the Animal Welfare and Ethics Committee of the Institute of Chinese Academy of Agricultural Sciences (permit number SCXK‐2021‐0006).

### Purification of SPJ

*Schisandra chinensis* (Turcz.) Baill was obtained and identified by the ‘Engineering Research Center for Glycocomplexes of the Ministry of Education’, College of Life Sciences of Northeast Normal University, and the ripe fruits were boiled and extracted twice with boiling water. The extract was precipitated in 75% ethanol to obtain total polysaccharide. Total polysaccharides were fractionated by using DEAE‐cellulose ion exchange column chromatography to obtain neutral sugars and acidic sugar components. Acidic sugars were further fractionated by using DEAE‐cellulose ion exchange column chromatography by elution with 0.1 m NaCl, 0.2 m NaCl, and 0.3 m NaCl to yield three components: WSCPA‐1, WSCPA‐2, and WSCPA‐3. The meaning of WSCPA is an acid polysaccharide in water‐soluble *Schisandra chinensis* polysaccharide (W: water‐soluble, SC: *Schisandra chinensis*, P: polysaccharides, A: acidic polysaccharide). WSCPA‐2 was further fractionated by using gel column chromatography to obtain the WSCPA‐2a fraction (SPJ) for subsequent studies.

### Cell culture, cytotoxicity, and virus infection assay

Cells were cultured in DMEM containing 5% or 10% heat‐inactivated fetal bovine serum (F8318, Sigma‐aldrich, Burlington, MA, USA) mixed with 1%  (P4333, Sigma‐aldrich) at 37 °C in a 5% CO_2_ incubator. Cell monolayers at 80% density (1 × 10^5^ cells) were used for viral inoculation and cytotoxicity assays. Different concentrations of SPJ were added to cells. After 36 h, 10 μL MTT (5 mg·mL^−1^) was added to each well and incubated at 37 °C for 4 h. Before measuring the optical absorbance (OD_570_), 100 μL DMSO was added to dissolve formazan crystals for 10–15 min. For the viral infection experiment, viral strains were passaged and inoculated into cells at a selected multiplicity of infection (MOI = 0.1) that were supplemented with 5% L‐1‐Tosylamido‐2‐phenylethyl chloromethyl ketone (TPCK) treated trypsin. MOI refers to the ratio of virus to number of cells during infection; the calculation formula is: viral titers/number of cells. The morphology of MDCK and A549 cells was observed before performing the MTT assay. Select blank cells as the positive control and virus wells as the negative control for each experiment. Set up six duplicate wells and repeat each experiment for three times. The calculation formula of inhibition rate is: Inhibition rate (%) = (drug treatment OD − virus control OD) ÷ (cell control OD − virus control OD) × 100%. Results were analyzed using graphpad prism 8.0, and the half inhibitory concentration (IC_50_) and half maximum effective concentration (EC_50_) of SPJ on cells were calculated.

To examine the effects of SPJ on influenza viruses at different stages of the life cycle, the MDCK and A549 cells were treated in three different strategies, including pretreatment, co‐treatment, and post‐treatment. Pretreatment: H1N1‐UI182 virus was preincubated with SPJ for 1 h at 4 °C and subsequently used for infection. The cells were infected with the complex of H1N1 virus and SPJ polysaccharide for another 1 h at 37 °C. Co‐treatment: MDCK and A549 cells were exposed to DMEM containing the virus and SPJ for 1 h at 37 °C. Post‐treatment: the cells were infected with H1N1‐UI182 virus in the absence of SPJ. After viral adsorption occurred for 1 h at 37 °C, the non‐adherent viruses were removed, and the cells were washed twice and subsequently incubated with DMEM containing SPJ.

### RNA extraction and real‐time PCR

Lung tissues or cells were lysed using the HiPure Universal RNA Kit (Shanghai, China), and total RNA was extracted. Then 1 μg template RNA was reverse‐transcribed to cDNA by using Perfect Real Time (Otsu, Japan). Primer sequences are shown in Table S1, and *GADPH* was used as a housekeeping gene for data normalization.

### Immunofluorescence assay

A549 cells were fixed with paraformaldehyde solution (4% PFA), permeabilized with 0.2% Triton‐X100, and blocked with 2% BSA (Shanghai, China) for 1 h. Primary antibody against influenza virus nucleoprotein (vNP: 1 : 200) was prepared using 5% BSA and incubated overnight at 4 °C. After three washes with PBS, a mouse‐red secondary antibody (Shanghai, China) mixed with 5% BSA as a maker, and nuclei were stained with tetrahydrate (dibenzidine, DAPI, 10 μg·mL^−1^) at room temperature and incubated for 10 to 20 min. Fluorescence was detected using a fluorescence microscope (Axio Vert A1, Carl Zeiss, Oberkohen, Batenwerburg, Germany).

### *In vivo* experiments in mice

Six‐to‐eight‐week‐old female BALB/c mice (18–20 g) were obtained from Vital River Laboratory Animal Technology Co., Ltd. (Beijing, China). Mice were housed in Type II RVC‐independent air supply isolation cage for at least 1 week before starting the experiment, and fed with double distilled water and mouse food (SPF‐F02‐001, Beijing, China). Subsequently, they were randomly divided into four groups: normal control group in an untreated and uninfected condition (Control), model group (Virus), Virus + SPJ (Abdomen) group (2.5 mg·kg^−1^·day^−1^), and Virus + SPJ (Stomach) group (2.5 mg·kg^−1^·day^−1^), *n* = 13 in each group. The mLD_50_ which is the virus titers of mice at half lethal dose of H1N1‐UI182 is 1 × 10^4.37^, and we use DMEM to dilute the influenza virus. Except the control group, other groups were intra‐nasally inhaled with 15 mLD_50_ per 50 μL lethal dose of viral solution after anesthesia to establish the infection model. Following 12 h of infection, drug intervention was performed twice daily (morning and evening) for 5 consecutive days, and body weight was recorded at the same time each day. On 3 day postinfection (3 dpi) and 5 dpi, three mice in each group were randomly chosen to collect arterial blood and euthanized to dissect the organs (lung, heart, liver, spleen, and kidney). After 2 h at room temperature, centrifuged the blood samples to collect serum, and stored the serum at −80 °C or directly used it for ELISA (enzyme‐linked immunosorbent assay). The lung, heart, liver, spleen, and kidney were fixed with 4% PFA and then detected with hematoxylin–eosin (HE) staining to observe the pathological damage. Subsequently, lung weight was recorded and lung index was assessed. The lung index can be used as an important indicator to judge the severity of lung tissue injury. The calculation formula for lung index is: (lung weight/body weight) × 100%. The body weight of the remaining mice and the survival status in each group were measured daily until 9 dpi.

### Virus titers and hemagglutination inhibition assay

On 3 and 5 dpi, the lung tissues of infected mice in each group after euthanization were collected. Then ground it with DMEM and obtained the supernatant after centrifugation. The supernatant was serially diluted from 10^−1^ to 10^−8^ and inoculated the dilutions into three 9‐day‐old SPF chicken embryos. After 72 h of incubation at 37 °C, we mixed 50 μL of chicken embryos' allantoic fluid with 50 μL of chicken blood containing 1% red blood cells. After incubation at room temperature for 30 min, changes in the level of hemagglutination were observed, viral titers were calculated by using the Reed‐Muench method, and the results were expressed as log_10_EID_50_.

### Pulmonary pathology experiment

Three mice were randomly chosen and euthanized, and the lung, heart, liver, spleen, and kidney tissues were quickly placed in 4% PFA for 48–72 h for fixation. After this, tissues were embedded in paraffin and randomly cut into 4–8 μm thin slices. Sections were then dewaxed with xylene and soaked in absolute ethanol for 10 to 20 min. Finally, sections were stained with hematoxylin and eosin, dried, and then observed under a light microscope. The pathological severity score of the infected mice was based on the percentage of the inflammation area of each slice collected from each animal, and using the following scoring system: 0, no pathological changes; 1, affected area ≤ 10%; 2, affected area < 50% and > 10%; and 3, affected area is ≥ 50%. When alveolar wall thickening, inflammation, congestion, bleeding, and bronchial necrotic cell fragments were observed, the score will be increased by one point.

### Immunohistochemical experiments

Lung tissues were de‐paraffinized in xylene and then dehydrated in ethanol. Endogenous peroxidase activity was blocked by treatment with 0.3% H_2_O_2_ in methanol for 20 min at room temperature. After antigen retrieval, tissue sections were blocked with 5% goat serum for 20 min at room temperature, and then with anti‐vNP antibody which is purchased from Abcam Company overnight at 4 °C. After washing, tissue slides were incubated with biotinylated secondary antibodies (Shanghai, China) for 1 h at room temperature, then stained with diaminobenzidine (DAB), and counterstained with hematoxylin. Finally, slides were evaluated under the microscope.

### Enzyme‐linked immunosorbent assay (ELISA)

Serum samples from different groups of mice were collected on 5 dpi, and the concentration of cytokines in the samples was determined according to the manufacturer's instructions of mouse TNF‐α ELISA kit (JM‐02415M1), mouse IL‐1β ELISA kit (JM‐02323M1), mouse IFN‐γ ELISA kit (JM‐02465M1), and mouse IL‐6 ELISA kit (JM‐02446M1) purchased from Jingmei Corporation (Jiangsu, China). The kit adopts the double‐antibody sandwich method. Blank well, negative control, positive control, and duplicate well are set for each experiment, and the results are compared with the standard curve.

### Statistical analysis

Results were analyzed using graphpad prism 8.0 (La Jolla, CA, USA) and presented as the mean ± standard error (SE). Statistical significance was determined using ANOVA analysis. *P*‐values < 0.05 were taken to indicate significant differences, *P < 0.05, **P < 0.01, ***P < 0.0001.

## Results

### Extraction and identification of SPJ

**Fig. 1:**
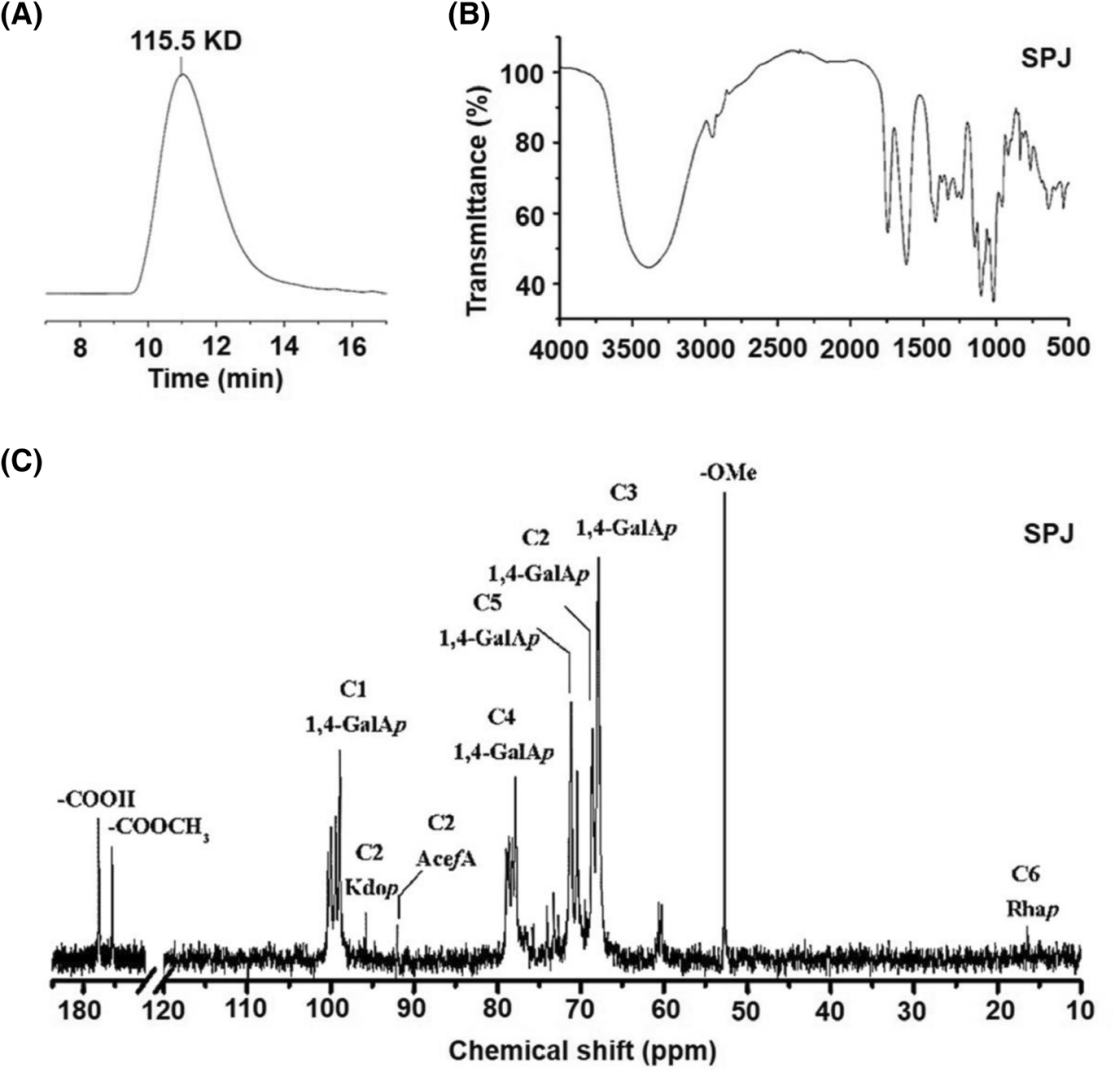
Extraction and structure analysis of *Schisandra* polysaccharide (SPJ). (A) The Molecular weight distribution of SPJ by high‐performance gel permeation chromatography. (B) The structure of SPJ determined by infrared spectroscopy. (C) PNMR spectrum of the glycosidic bond configuration of each component of SPJ.

Antiviral activity of SPJ is related to the spatial structure of polysaccharide. Thus, we examined the composition of SPJ, as shown in Table 1. By gel permeation chromatography, the averaged molecular weight of SPJ was determined to be 115.5 KD (Fig. 1A). The structure and functional groups of the polysaccharides were determined and analyzed by infrared spectroscopy in which we identified, for example, characteristic absorption peaks known for polysaccharides at 3000 to 3600 cm^−1^, 1500 to 1650 cm^−1^, and 875 to 1200 cm^−1^. We also noted a stretching vibration peak for C–O–C and C–O–H for the characteristic configuration of pyranose rings at 1000 to 1200 cm^−1^, a stretching vibration peak for the N=N bond at 1575 to 1630 cm^−1^, a stretching vibration peak for C=O at 1645 to 1690 cm^−1^, and a stretching vibration peak for OH at 3000 to 3750 cm^−1^ (Fig. 1B). Using ^1^H nuclear magnetic resonance (PNMR) spectroscopy, we observed that SPJ mostly contains highly methylated homogalacturonic acid (HG)‐type pectin domains formed by linkage of α‐1,4‐GalA residues and galacturonic acid (GalA) that occurs to the same degree as methyl esterification (Fig. 1C).

**Table 1:** The composition of *Schisandra* polysaccharide (SPJ).

Composition	Monosaccharide composition (%)				
	Galactonic acid	Rhamnose	Galactose	Arabinose	Glucose
SPJ	89.4	0.8	4.4	3.8	1.7

### SPJ has inhibitory activity against influenza virus infection

**Fig. 2:**
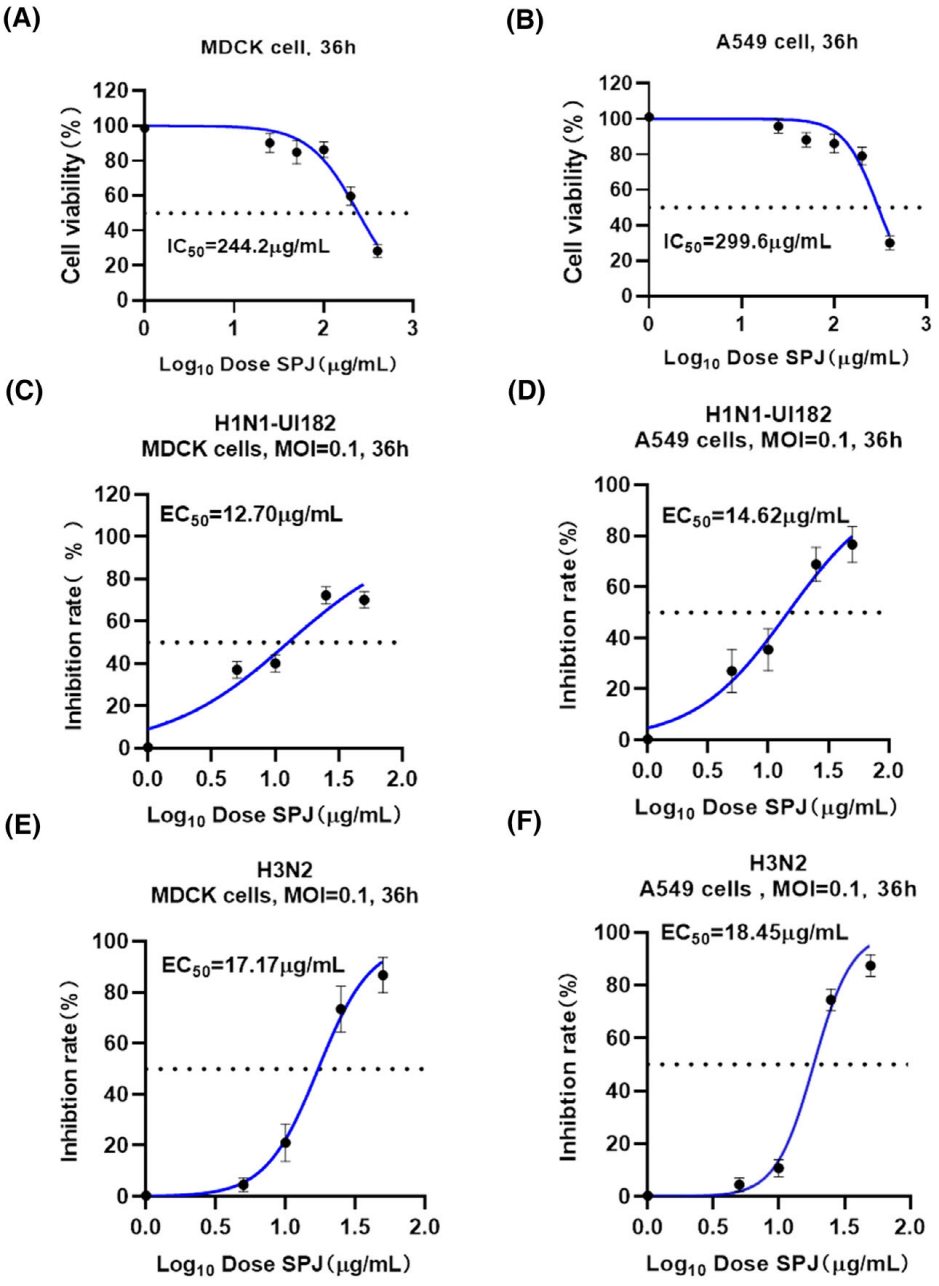
Inhibitory effect of *Schisandra* polysaccharide (SPJ) on influenza virus *in vitro*. (A) The toxic effects of SPJ on cell growth of MDCK cells. (B) The toxic effects of SPJ on cell growth of A549 cells. (C) The antiviral activity of SPJ against influenza virus H1N1 in MDCK cells. (D) The antiviral activity of SPJ against influenza virus H1N1 in A549 cells. (E) The antiviral activity of SPJ against influenza virus H3N2 in MDCK cells. (F) The antiviral activity of SPJ against influenza virus H3N2 in A549 cells. Data represent mean ± SD, *n* = 3 independent experiments.

To evaluate whether SPJ has anti‐influenza viral activity, we first examined the dose‐dependent relationship between SPJ and cell growth inhibition in MDCK and A549 cells. Our results show that SPJ inhibits cell growth in a dose‐dependent manner, with a median inhibitory concentration (IC_50_) of MDCK at 244.2 μg·mL^−1^ (Fig. 2A) and A549 at 299.6 μg·mL^−1^ (Fig. 2B). We next examined the antiviral activity of SPJ on H1N1 and H3N2 infected cells. Antiviral activity of SPJ against influenza virus was measured by viral replication in cultures exposed to different doses of SPJ for 36 h. Results show that SPJ has effective antiviral activity at the mean half‐maximal effective concentration (EC_50_), with EC_50_ values of 12.7 μg·mL^−1^ for H1N1 (Fig. 2C) and 17.17 μg·mL^−1^ for H3N2 (Fig. 2E) in MDCK cells, and with EC_50_ values of 14.62 μg·mL^−1^ for H1N1 (Fig. 2D) and 18.45 μg·mL^−1^ for H3N2 (Fig. 2F) in A549 cells. Overall, our results indicate that SPJ has effective antiviral activity in human cell lines.

**Fig. 3:**
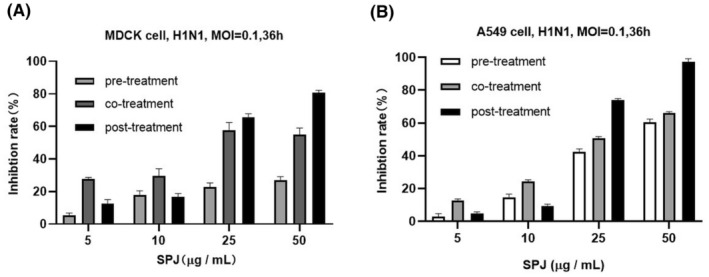
Effects of different administration modes of *Schisandra* polysaccharide (SPJ) on influenza virus. (A) The anti‐influenza viral activity stage of *Schisandra* polysaccharide SPJ was clarified by using different administration routes in MDCK cells. (B) The anti‐influenza viral activity stage of SPJ was determined by using different routes of administration in A549 cells. Data represent mean ± SD, *n* = 3 independent experiments.

**Fig. 4:**
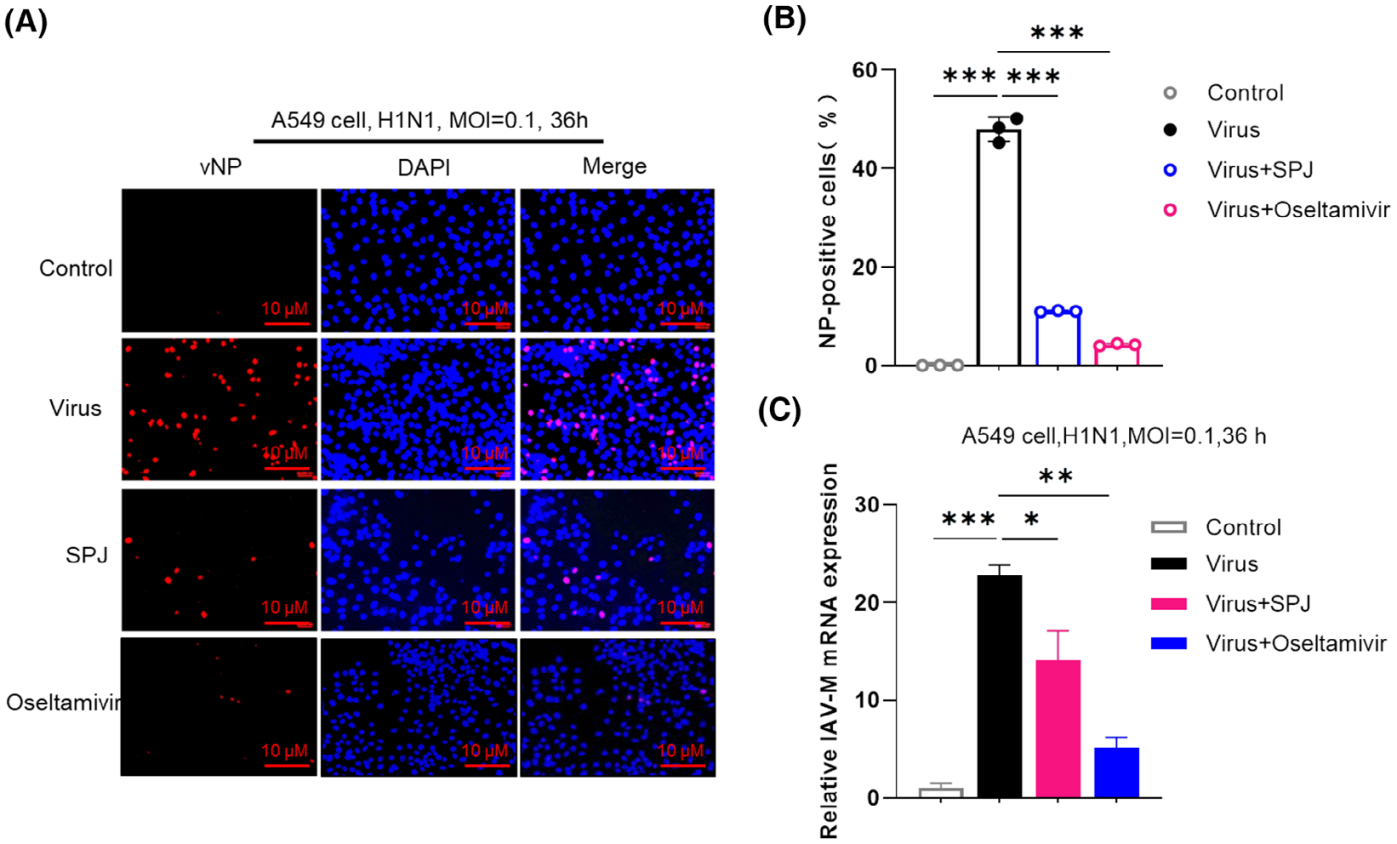
*Schisandra* polysaccharide (SPJ) inhibits influenza viral replication in cells. (A) Immunofluorescence assay to detect the expression of viral nucleoprotein vNP, the scale bar is 10 μm. (B) The numbers of vNP‐positive nuclei were quantified in (A). (C) The mRNA expression of influenza virus M gene after SPJ treatment by RT‐qPCR, using GADPH as a control. Data represent mean ± SD, *n* = 3 independent experiments, and ANOVA analysis was used for multiple groups. *P < 0.05, **P < 0.01, ***P < 0.001.

To clarify the active stage of SPJ against influenza virus, we treated MDCK and A549 cells using three strategies of administration, that is, pretreatment, co‐treatment, and post‐treatment. Post‐treatment with SPJ exhibited a relatively high inhibition rate in MDCK (Fig. 3A) and A549 cells (Fig. 3B) with strong antiviral activity at 50 μg·mL^−1^. Cell morphology was captured before calculating IC_50_, which is 36 h after virus inoculation and 24 h after SPJ treatment. The results indicated the absence of cytotoxicity in uninfected cell cultures treated with similar doses of SPJ (Fig. S1A,B). Therefore, we chose this concentration for subsequent experiments. To further explore antiviral effects of SPJ, we chose oseltamivir as a positive control, which was commonly used to treat viral infections. The appearance of vNP proteins in infected cells was quantified by fluorescence detection (Fig. 4A). As expected, oseltamivir had a significant inhibitory effect on influenza virus, and the concentration of it was 100 μg·mL^−1^ in the experiments. Furthermore, we found that 48% of A549 nuclei were vNP‐positive in the DMSO‐treated control group, whereas the percentage of vNP‐positive cells decreased to 11% upon SPJ treatment (Fig. 4B). Interestingly, SPJ treatment significantly decreased viral RNA copy number in A549 cells (Fig. 4C). Our findings suggest that SPJ treatment significantly inhibits influenza virus replication *in vitro*, with the inhibitory effect being positively correlated to the dose of SPJ.

### Therapeutic effect of SPJ on influenza virus‐infected mice

**Fig. 5:**
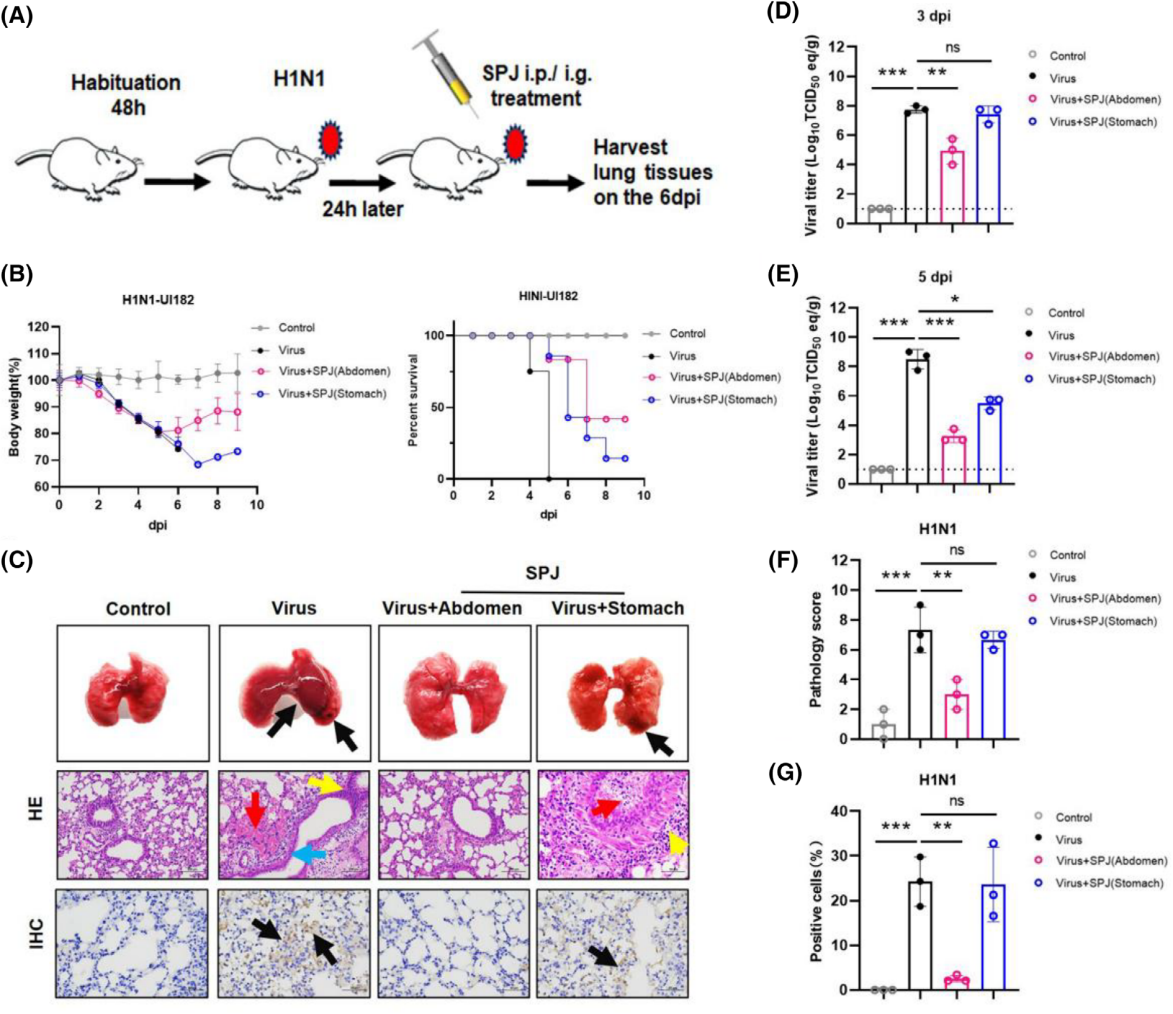
Inhibitory effect of *Schisandra* polysaccharide (SPJ) on IAV *in vivo*. (A) Construction and treatment simulation diagram of virus infection model mice. (B) The average body weight change rate and survival curves of mice in the control group (control), the H1N1 virus infection model group (Virus), intraperitoneal injection treatment group (Virus + SPJ (Abdomen)), and gavage treatment group (Virus + SPJ (Stomach)). (C) Morphological observation, HE staining, and pathological observation of mice after exposure and treatment, the scale bar is 50 μm. (D, E) Virus titers in lung tissues of the four groups. (F) Pathology score results of HE were statistically analyzed. (G) The number of vNP‐positive cells was counted by IHC. Data represent mean ± SD, *n* = 3 independent experiments, and ANOVA analysis was used for multiple groups. *P < 0.05, **P < 0.01, ***P < 0.001.

To evaluate the therapeutic effect of SPJ *in vivo*, we used a mouse model infected with influenza virus. Considering the absorption route of SPJ *in vivo*, we administered the compound to mice by gavage and by intraperitoneal injection (Fig. 5A). The body weight of mice began to decrease on the second day of viral exposure (Fig. 5B), and mice succumb to the infection on the fourth day, and all had succumbed by the fifth day. However, mice treated with SPJ remained alive on the ninth day following treatment, with intraperitoneal treatment being more effective than oral treatment (Fig. 5B). Overall, our findings indicate that SPJ treatment prolongs the survival time of infected mice.

The lung index can be used as an important indicator to judge the severity of restrictive lung diseases [26]. Therefore, we analyzed changes in the lung index of mice prior to SPJ administration, as well as on 3 and 5 dpi, and we observed that SPJ abdomen treatment reduced an increase in the lung index after viral infection (Fig. S2A,B). Indeed, viral infection caused severe pathological symptoms, which could be reversed by SPJ abdomen treatment (Fig. 5C). Detection of viral titers in the lungs showed that abdomen treatment could significantly reduce viral titers in lung tissues of infected mice (Fig. 5D,E). Pathological analysis showed that viral infection caused massive hemorrhaging in lung tissues (Fig. 5C red arrow) and narrowing of trachea (Fig. 5C blue arrow) that was accompanied by increased infiltration of inflammatory cells (Fig. 5C yellow arrow). In addition, after virus infection, the spleen showed a small amount of slight congestion and expansion of the medullary sinus (Fig. S3 black arrow), and necrosis of renal tubular epithelial cells and irregular shape of glomeruli appeared in the kidney (Fig. S3 green arrow). SPJ abdomen treatment significantly improved these phenomena (Fig. 5C, Fig. S3), and histopathological score indicated that SPJ effectively attenuates pathological damage caused by viral infection (Fig. 5F). In addition, immunohistochemistry (IHC) showed that the drug significantly inhibited the expression of vNP proteins in lung tissues (Fig. 5G). Our results indicate that SPJ has a positive protective and therapeutic effect on cells/tissues infected with influenza virus.

### SPJ treatment reduces the ‘cytokine storm’ caused by influenza viral infection

**Fig. 6:**
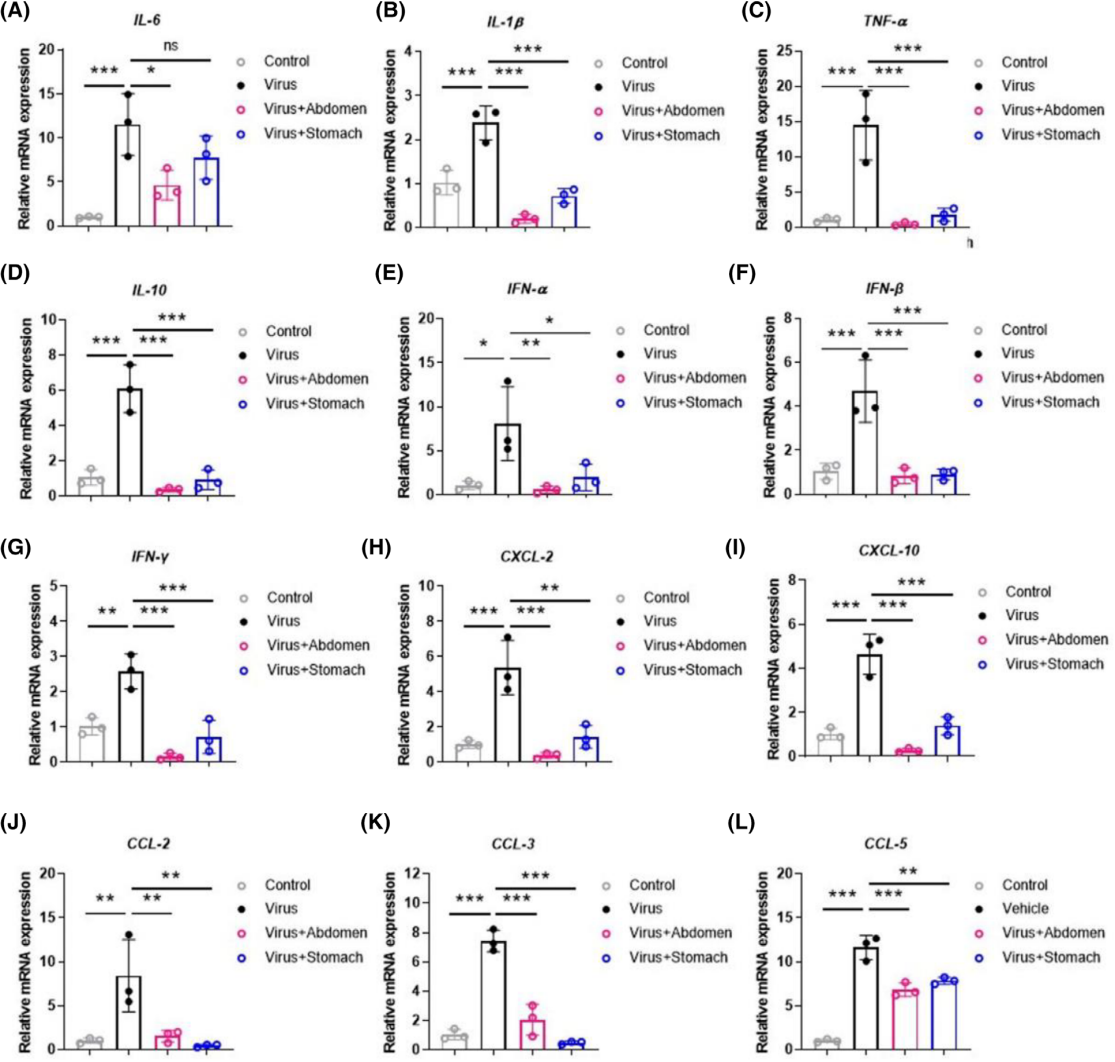
*Schisandra* polysaccharide (SPJ) inhibits the ‘cytokine storm’ induced by influenza viral infection on 5 dpi. (A‐L) IL‐6, IL‐1β, TNF‐α, IL‐10, IFN‐α, IFN‐β, and IFN‐γ, CXCL‐2, CXCL‐10, CCL‐2, CCL‐3, and CCL‐5. Data represent mean ± SD, *n* = 3 independent experiments, and ANOVA analysis was used for multiple groups. *P < 0.05, **P < 0.01, ***P < 0.001.

**Fig. 7:**
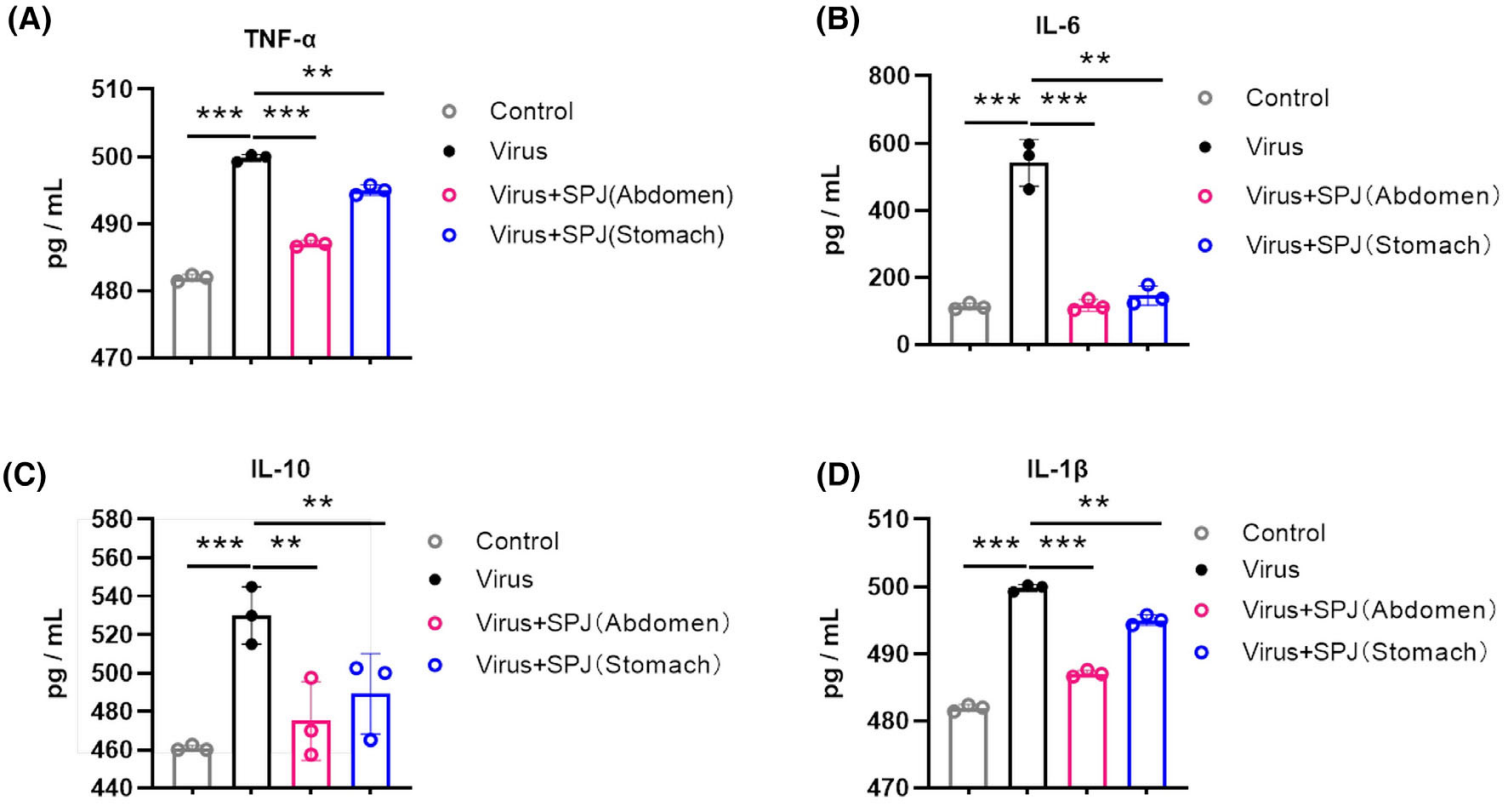
*Schisandra* polysaccharide (SPJ) inhibits inflammation‐associated cytokine levels in serum on 5 dpi. (A) TNF‐α, (B) IL‐6, (C) IL‐10, (D) and IL‐1β. Data represent mean ± SD, *n* = 3 independent experiments, and ANOVA analysis was used for multiple groups. **P < 0.01, ***P < 0.001.

Previous studies have shown that influenza viral infection causes amplification of the cytokine storm in mice [27]. To explore the effect of SPJ treatment on cytokine expression, we used real‐time PCR and observed that viral infection led to increased expression of cytokines and chemokines, including *IL‐6*, *IL‐1β*, *TNF‐α*, *IL‐10*, *IFN‐α*, *IFN‐β*, and *IFN‐γ*, as well as *CXCL‐2*, *CXCL‐10*, *CCL‐2*, *CCL‐3*, and *CCL‐5* (Fig. 6A–L). However, this ‘storm’ could be reversed by treatment with SPJ. In addition, similar results were obtained by detecting protein concentration of inflammatory factors in serum, such as *TNF‐α*, *IL‐6*, *IL‐10*, and *IL‐1β* (Fig. 7A–D). Our results suggest that SPJ attenuates the cytokine storm in infected mice and reduces the inflammatory response.

## Discussion

Viral infection seriously threatens human health. Respiratory system viral infections can cause mild, severe, or even fatal disease [28]. These viruses not only induce respiratory and systemic symptoms, but also exhibit characteristics of rapid replication [29], which has brought serious challenges to global public health. Influenza virus is a zoonotic pathogen with a wide host range and is difficult to prevent and control [30]. Therefore, the ability to effectively prevent and control influenza has become the primary global for public health on the world stage. Despite numerous studies with vaccines and drug developments against IAV, current treatments of IAV infection are mostly conservative [31]. In the present study, we have investigated the role of the natural plant polysaccharide SPJ in the treatment of IAV infection and found it to be highly effective against influenza viral infection and not toxic to normal cells.

*Schisandra chinensis* contains a variety of organic components, mainly lignans, volatile oils, polysaccharides, organic acids, fatty acids, proteins, etc. [32]. These compounds have various activities, such as immune regulation, liver protection, antifatigue, antioxidation, antitumor effects, and functional improvement of the reproductive system [33]. As a polysaccharide extract from *Schisandra chinensis*, SPJ can improve liver fibrosis, with clear protective effects on liver cells [34]. Notably, administration of SPJ can modulate cytokine expression in the serum of immunosuppressed [35], which suggests that SPJ may play an important role in cytokine‐induced immune responses and related diseases. As a kind of natural polysaccharide, SPJ is composed of monosaccharides (galacturonic acid, rhamnose, galactose, arabinose, and glucose) in different proportions, and its pharmacokinetics is relatively complex. In addition, the proper choice of drug delivery mode is critical to the bioavailability of drugs. Oral administration by gavage enters the blood circulation after absorption through the stomach and intestines, and intraperitoneal injection is absorbed through the peritoneum, with a large absorption area, and directly enters the blood. In this study, we found that the therapeutic effect of intraperitoneal injection is better than that of oral administration, and SPJ has protective effects on lung tissues in mice by reducing viral load upon infection. Importantly, SPJ administration improves survival upon lethal infection with influenza virus and suppresses the inflammatory response promoted by inactivation of the cytokine storm.

Viral infection, especially which from the highly pathogenic influenza virus, leads to a marked increase in pro‐inflammatory cytokines that induce the ‘cytokine storm’ hypothesized to be the main cause of host death [36]. Previous studies have shown that mortality from influenza viral infection can be reduced by inhibiting cytokines that regulate inflammatory messages [37]. In addition, the ‘cytokine storm’, characterized by overproduction and dysfunction of inflammatory cytokines, has become an important cause of host death during influenza viral challenge [38], an effect that is used as a predictor of poor prognosis [39]. Importantly, we demonstrated here that SPJ significantly reduces mortality in influenza virus‐infected mice by inhibiting the ‘cytokine storm’. Our findings suggest that SPJ has therapeutic properties against IAV infection, and future studies could focus on whether SPJ has effect on other diseases caused by inflammatory reaction, so as to further study the specific mechanism of SPJ.

As we all know, the antiviral mechanism of plant‐derived polysaccharides is a complex process. The mechanism of polysaccharides with antiviral activity may have the following three aspects. First, polysaccharides directly targets host cells to interfere with virus infection [40]. Second, polysaccharides induce the expression of related host antiviral proteins, that is, intracellular signaling pathways [41]. Pretreatment of IAV with fucoidan KW may interferes with the activation of EGFR pathway to resist virus infection [42]. Third, polysaccharides have the ability of immune regulation, for instance; the immunoregulatory activity of *Schisandra chinensis* polysaccharide has been explored [43]. Here, our *in vitro* experiments confirmed that the key time of SPJ action is the stage of postadsorption of virus infection. The steps in the IAV life contain cycle viral entry, viral RNA transcription and replication, protein expression, and viral budding [44]. Post‐treatment with SPJ exhibits a relatively high inhibition rate; these results indicate that SPJ has significant inhibitory effects on the replication and release of influenza virus H1N1‐UI182 *in vitro*.

## Conclusion

In conclusion, SPJ has been found to have inhibitory properties, which can effectively inhibit IAV proliferation in cell culture models without cytotoxicity. Furthermore, SPJ treatment results in a decrease in mortality and a significant increase in survival upon infection with influenza virus. Therefore, we propose that SPJ may be a potential therapeutic agent against influenza viral infection. Future studies will be aimed at examining the effects of SPJ on other respiratory viruses and other strains of influenza.

## Conflict of interest

The authors declare no conflict of interest.

### Peer review

The peer review history for this article is available at https://www.webofscience.com/api/gateway/wos/peer‐review/10.1002/2211‐5463.13690.

## Author contributions

JQ contributed to the data analysis, writing—original draft, performing the experiments, and writing—review and editing. JG contributed to the writing—original draft and preparation of figures. CL contributed to the data curation, writing—original draft, methodology, software, and writing–review and editing. YL and MS provided technical support for experiments. RL contributed to the reparation of figures and tables and performed the experiments. HX contributed to the data analysis and performed the experiments. XX contributed to the supervision and project administration. YZ contributed to the supervision, writing—review and editing, and purified and identified *Schisandra* polysaccharide (SPJ) from *Schisandra chinensis*. TW contributed to the supervision, funding acquisition, and writing—review and editing.

## Supporting information


**Fig. S1.** Effects of SPJ treatment on cell growth and morphology with or without H1N1 infection.
**Fig. S2.** SPJ treatment shows protective effects on lung tissue in infected mice.
**Fig. S3.** Effects of SPJ treatment on the pathology of heart, liver, spleen, and kidney tissue in IAV‐infected mice.
**Table S1.** Primer sequences for PCR.

## Data Availability

All the data generated during the current study are included in the manuscript.
