# Supplementary material for: Schisandra chinensis (Turcz.) Baill. polysaccharide inhibits influenza A virus in vitro and in vivo
Source: FEBS Open Bio. 2023 Sep 1;13(10):1831–43. doi: 10.1002/2211-5463.13690 (PMC10549229; doi:10.1002/2211-5463.13690)
Supplement: Supplementary file 1 — Fig. S1. Effects of SPJ treatment on cell growth and morphology with or without H1N1 infection. Fig. S2. SPJ treatment shows protective effects on lung tissue in infected mice. Fig. S3. Effects of SPJ treatment on the pathology of heart, liver, spleen, and kidney tissue in IAV‐infected mice. Table S1. Primer sequences for PCR. [file FEB4-13-1831-s001.doc]

**Tables**

**Table S1, Primer sequences for PCR**

| Gene Name | Primer Sequence (5’ to 3’) |
| --- | --- |
| *M* | F: 5’-CACACACGTCTCCGGGAGCAAAAGCAGGTAG-3’ |
|  | R: 5’-CACACACGTCTCCTATTAGTAGAAACAAGGTAGTTTTT-3’ |
| *GAPDH* | F: 5’-CACCATGGAGAAGGCCGGGGCCCAC-3’ |
|  | R: 5’-ATCATACTTGGCAGGTTTCTCCAGG-3’ |
| *IFN-α* | F: 5’-GCACCCTGCCTCAGACTCAC-3’  R: 5’-TGCCTGGTCATCTCATGGAAG-3’ |
| *IFN-β* | F: 5’-GCTGGTATATCCTCCAAATCGC-3’  R: 5’-CCCAAGACGTTCTGAAGCATC-3’ |
| *IFN-γ* | F: 5’-AGCCAAATCGTCTCCTTCTACTTC-3’  R: 5’-TGCACCTTGTTGCTGCTGTT-3’ |
| *TNF-α* | F: 5’-AGCCCTGGTATGAACCCATC-3’  R: 5’-GGAATCGGCAAAGTCAAGGT-3’ |
| *IL-1β* | F:5’-TCATCGTGGCAGTGGAAAAG-3’  R: 5’-GGGAAGCAAGGGTCTCAGGT-3’ |
| *IL-6* | F: 5’-AGTTGCCTTCTTGGGACTGATG-3’  R: 5’-GGGAGTGGTATCCTCTGTGAAGTCT-3’ |
| *IL-10* | F:5’-TGACCCAGACATCAAGGAACAT-3’  R: 5’-GTCAAACTCACTCATGGCTTTGTA-3’ |
| *CCL2* | F: 5’- ACACAGAAGTGGGTGCAGGA-3’  R: 5’-GTTCTTGGGGTCTTGGGTTG-3’ |
| *CCL3* | F: 5’-GCTCAACATCATGAAGGTCTCC-3’  R: 5’-TGCCGGTTTCTCTTAGTCAGG-3’ |
| *CCL5* | F: 5’-CTCCTTGCTGCTTTGCCTAC-3’  R: 5’-ACACACCTGGCGGTTCTTTC-3’ |
| *CXCL-2* | F: 5’-CTCAAGAACATCCAAAGTGTG-3’  R: 5’-ATTCTTGAGTGTGGCTATGAC-3’ |
| *CXCL-10* | F: 5’- CAGCAGTCCGCAGTATAAACAGT-3’  R: 5’-GCCAAGTACCTAACGCTCACC-3’ |

**Fig. S1** Effects of SPJ treatment on cell growth and morphology with or without H1N1 infection. (A) Effects of SPJ treatment on growth and morphology of MDCK cells. (B) Effects of SPJ treatment on the growth and morphology of A549 cells. The scale bar is 10 µm.

**Fig. S2** SPJ treatment shows protective effects on lung tissue in infected mice. (A) The lung index of mice in each group on 3-dpi. (B) The lung index of mice in each group on 5-dpi. Data represents mean ± SD, n = 3 independent experiments, and ANOVA analysis was used for multiple groups. ** p <0.01, *** p <0.001, **** p <0.0001.

**Fig. S3** Effects of SPJ treatment on the pathology of heart, liver, spleen and kidney tissue in IAV-infected mice. Black arrows indicate apoptotic bodies and green arrows indicate glomerulus. The scale bar is 50 µm

**Fig. S1**


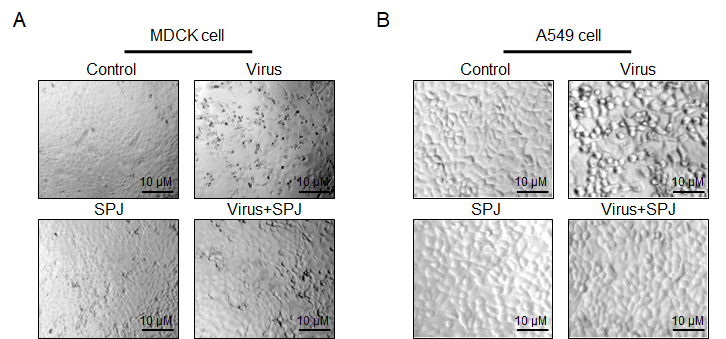


**Fig. S2**


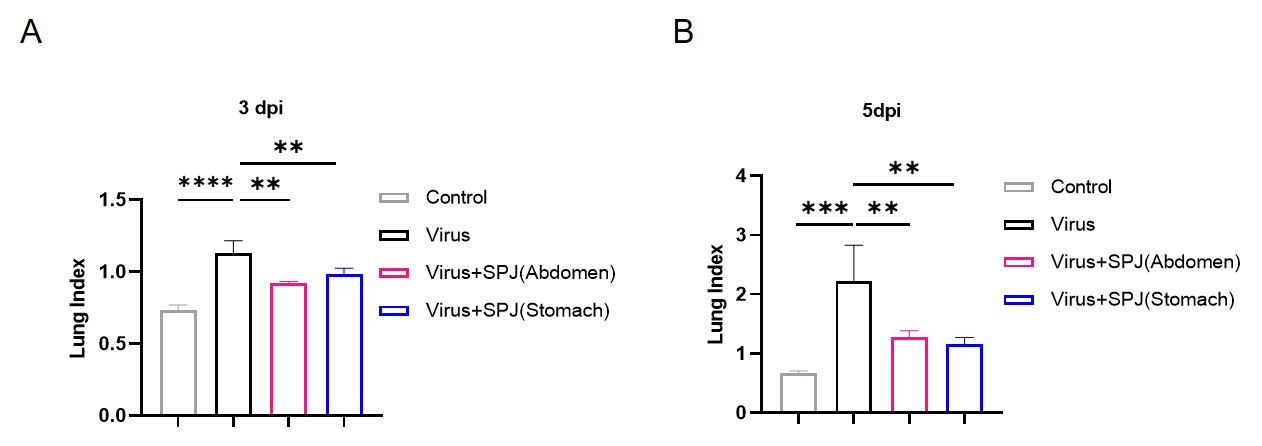


**Fig. S3**

**
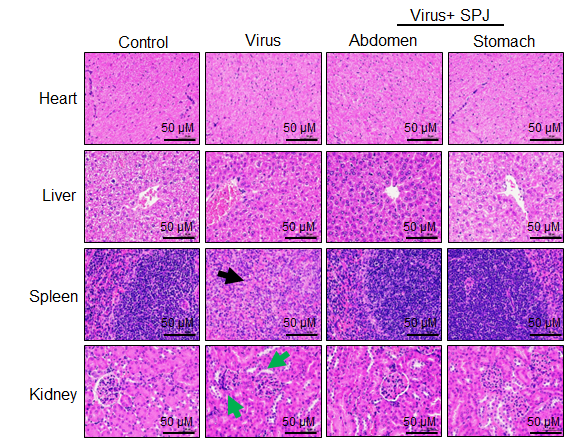
**
